# Chemical Composition and the Cytotoxic, Antimicrobial, and Anti-Inflammatory Activities of the Fruit Peel Essential Oil from *Spondias pinnata* (Anacardiaceae) in Xishuangbanna, Southwest China

**DOI:** 10.3390/molecules25020343

**Published:** 2020-01-15

**Authors:** Ren Li, Jing-Jing Yang, Xing-Zhen Song, Yuan-Fei Wang, Richard T. Corlett, You-Kai Xu, Hua-Bin Hu

**Affiliations:** 1 Southeast Asia Biodiversity Research Institute, Chinese Academy of Sciences & Center for Integrative Conservation, Xishuangbanna Tropical Botanical Garden, Chinese Academy of Sciences, Mengla 666303, Yunnan, China; corlett@xtbg.org.cn; 2 Chinese Academy of Sciences, Beijing 100049, China; songxingzhen@xtbg.ac.cn; 3 National Engineering Research Center for the Emergency Drug, Beijing Institute of Pharmacology and Toxicology, Beijing 100850, China; yangjingjing105@163.com; 4 CAS Key Laboratory of Tropical Plant Resources and Sustainable Use, Xishuangbanna Tropical Botanical Garden, Chinese Academy of Sciences, Mengla 666303, Yunnan, China; 5 Yunnan University of Traditional Chinese Medicine, Kunming 650500, China; atalice@163.com (Y.-F.W.); xyk@xtbg.ac.cn (Y.-K.X.)

**Keywords:** *Spondias pinnata*, essential oil, antimicrobial, anti-inflammatory, *Aspergillus fumigatus*, Xishuangbanna

## Abstract

*Spondias pinnata* (Linn. f.) Kurz (Anacardiaceae) is widely distributed in tropical Asia, where it is commonly used as a vegetable and fruit, and is attracting increasing research attention. In this study, we investigated the chemical composition and the cytotoxic, antimicrobial, and anti-inflammatory activities of the fruit peel essential oil of *S. pinnata* (EOSP), which has been consumed as a medicine and condiment in Xishuangbanna, southwest China. A total of 40 components were identified by Gas Chromatography/Mass spectrometry (GC-MS), representing 95.19% of the EOSP, with furfural (17.14%), α-terpineol (13.09%), and ethyl benzoate (9.05%) as the main constituents. EOSP has moderate cytotoxic activity against five cancer cells and obvious antimicrobial activity against five pathogenic strains. In particular, EOSP (Minimal Inhibitory and Fungicidal Concentration, MIC and MFC, 16‒32 µg/mL) showed a 32-times higher inhibition effect against *Aspergillus fumigatus* than the positive control Tigecycline (MIC and MBC 512‒1024 µg/mL). EOSP also showed strong anti-inflammatory activity by significantly inhibiting nitric oxide (NO) production induced by lipopolysaccharide (LPS) in RAW 264.7 cell lines at 0.08‰, with no effect on cell viability. These bioactivities of *S. pinnata* fruit peel validate its traditional uses and suggest that it could be a new source of natural antimicrobial and anti-inflammatory agents for food or medical industries.

## 1. Introduction

Essential oils, which are a mixture of numerous fragrant compounds collected from aromatic plants, have been frequently utilized as an ingredient in pharmaceutical, antiseptic, household, and cosmetic products since ancient times. Essential oils and aromatic compounds from spices and aromatic herbs have been demonstrated to have various bioactivities, including antibacterial, antiviral, anti-inflammatory, antifungal, anti-tumor, and antioxidant, as well as other miscellaneous activities [1,2,3,4]. *Aspergillus fumigatus* is a widespread saprotroph in nature and also a major airborne fungal pathogen that can cause a wide range of infections in humans, including chronic lung disease and life-threatening systemic infections that can involve multiple organs [5]. More than 200,000 life-threatening infections each year are attributed to this pathogen and treatment has recently been further complicated by the global emergence of resistance to the triazole class of antifungals in both clinical and environmental isolates [6]. To overcome antifungal resistance and release the global burden of different infections, it is necessary to find new antimicrobial and anti-inflammatory agents.

The family Anacardiaceae has many economic uses in China, including species that are used as sources of fruits, nuts, timbers, lacquer, traditional medicines, tannins, dyes, vegetables, and seasonings [7]. Essential oils from some Anacardiaceae species have been demonstrated to have antioxidant, antimicrobial, and anti-inflammatory activities [8,9,10]. *Spondias pinnata* (Linn. f.) Kurz (Anacardiaceae) is a deciduous tree naturally distributed in southern Yunnan, China and also widely cultivated and naturalized in Bhutan, Cambodia, India, Indonesia, Laos, Peninsular Malaysia, Myanmar, Nepal, Philippines, Singapore, Thailand, and Vietnam [7]. It is widely used as both food and medicine throughout its range. For example, in Xishuangbanna, southwestern China, it is called “Ga li luo” and “Pai luo” by the local Dai and Aka people and its fruit and young leaves are consumed as a seasonal vegetable and wild fruit and made into a sour condiment [11,12,13]. The fruits and leaves are also utilized in Thailand, Malaysia, Nepal, and India and have been identified as a potential source of nutraceutical and flavoring agents because of their high content of flavonoids and phenolic compounds and their strong antioxidant and antimicrobial properties [14,15,16,17]. The fruit and stem bark have been used by the Dai ethnic group in Xishuangbanna as a traditional remedy for diarrhea, digestive disorders, whooping cough, detoxification, and the relief of swelling and pain [18,19]. The fruit is also used as an aromatic astringent and refrigerant for toning and treatment of rheumatic articular and muscular pain [20,21].

In the last decade, *S. pinnata* has attracted increasing research interest. Crude extracts of its fruit have been reported to have high nutrient value [14,22,23], antioxidant and antimicrobial activity [14,15], as well as hypoglycemic effects [24]. The leaf methanol extract and essential oil have also been shown to have significant antimicrobial activity [25,26,27] and the leaf ethanol extract was found to have strong antioxidant activity [28]. Extracts from *S. pinnata* bark exhibit anticancer activity against human glioblastoma [29] and have a potent protective effect against liver damage caused by both iron overload [30] and by ethanol [31], as well as having anti-inflammatory activity [32]. However, to the best of our knowledge, there is no information on the chemical composition and therapeutic-related bioactivity of the essential oil from its fruit peel.

**Figure 1:**
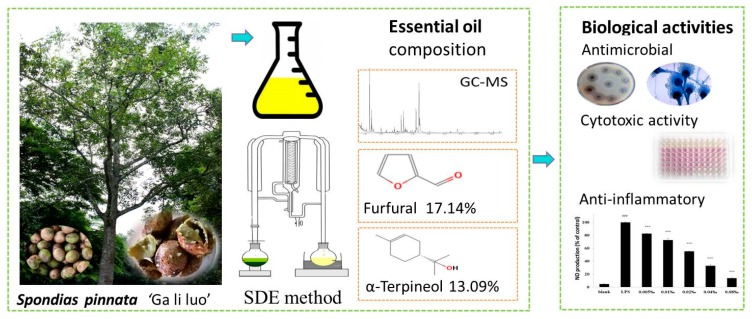
The main chemical components and the therapeutic related bioactivities of the essential oil from *Spondias pinnata* fruit peels. **SDE** means simultaneous distillation extraction.

In this study, we investigated the chemical composition and the cytotoxic, antimicrobial, and anti-inflammatory activities of the fruit peel essential oil of *S. pinnata* for the first time (Figure 1). *S. pinnata* is a fruit tree commonly cultivated for its high food and medicinal value in the home gardens of local villages in Xishuangbanna. This study aimed to produce scientific evidence in support of the traditional uses of *S. pinnata* as medicine and seasoning and, thus, support its potential application in medical and food industries.

## 2. Result and Discussion.

### 2.1. Essential Oil Composition

**Figure 2:**
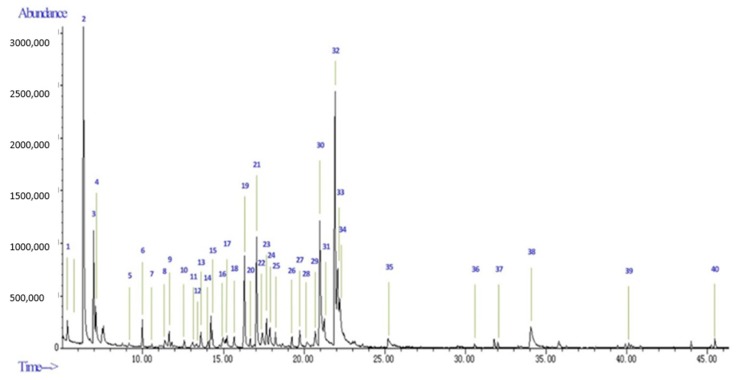
GC-MS chromatograms of the essential oil from the fruit peel of *Spondias pinnata*.

The yield of essential oil, collected by simultaneous distillation extraction from the fruit peels of *Spondias pinnata*, was found to be 0.05% (*w*/*w*) on a dry weight basis. A total of 40 components (Figure 2), representing 95.19% of the essential oil from *Spondias pinnata* (EOSP), were identified by Gas Chromatography/Mass spectrometry (GC-MS) (Table 1). EOSP was found to be rich in aliphatic alcohols (39.42%), monoterpene hydrocarbons (29.62%), and aromatics (22.03%), with furfural (17.14%), α-terpineol (13.09%), benzoic acid, ethyl ester (9.05%), methyl salicylate (5.88%), and γ-terpineol (5.55%) as the main components. A previous study of the volatile compounds of the whole green fruits of *S. pinnata* from the eastern region of India, isolated by a cryofocusing (cold trap) thermal desorption system (TDS) and GC-MS screening, showed that isopropyl myristinate (36.85%), isophorone (6.55%), limonene (4.46%), and linalool (3.57%) were the major compounds [14]. Investigation of the chemical composition of the hydro-distillated essential oil from the fruit of *S. pinnata* growing in Egypt showed the presence of long-chain alkanes, fatty acid, and fatty acid methyl esters with n-nonacosane (25.25%), α-terpineol (14.29%), and ethyl linolenate 11.72% as the main components [25]. These studies suggest that the main constituents of the essential oils from the whole green fruits, ripe fruits, and fruit peels of *S. pinnata* are different, although part of the differences could also be explained by differences in extraction methods and geographic locations. These differences could also partly explain the variation in reported bioactivities.

### 2.2. Cytotoxicity Activity

Essential oil of *Spondias pinnata* (EOSP) demonstrated an interesting cytotoxic activity against all tested human myeloid leukemia (HL-60), hepatocellular carcinoma (SMMC-7721), lung cancer (A-549), breast cancer (MCF-7), colon cancer (SW480), and human bronchial epithelial (BEAS-2B) cell lines, with IC_50_ values ranging between 13.26 and 50.21 µg/mL (Table 2). On one hand, EOSP cytotoxic activity was weaker than the positive control Cisplation, for which IC_50_ values ranged from 0.50 to 3.25 µg/mL. On the other hand, EOSP could still be a promising anticancer ingredient because it is easy to obtain from naturally cultivated trees and has a long history of medicinal use [19]. Furfural and its derivatives showed pronounced cytotoxicity activity in melanoma B16 cells with IC_50_ ranging from 10 to 32 µg/mL [33] and α-terpineol had potential anticancer effect in five human cancer cells through suppressing NF-κB [34]. These activities showed by the two main components of EOSP could partly explain its cytotoxicity. 

### 2.3. Antimicrobial Activity

The essential oil was active against all tested microbial strains with Minimal Inhibitory and Concentrations (MICs) ranging from 16 to more than 512 µg/mL (Table 3). The essential oil was extremely active against the tested fungi, including *Aspergillus fumigatus* and *Candida albicans*, with MICs ranging from 16 to 128 µg/mL and Minimal Fungicidal Concentrations (MFCs) ranging from 32 to 256 µg/mL. The essential oil showed a strong antifungal activity against *A. fumigatus*, with MIC/MFC values of 16–32 μg/mL, which are 32-times lower than those of the control Tigecycline, with MIC/MFC values of 512 to 1024 μg/mL, respectively. The search for new potential natural antimicrobial agents is a ceaseless task and solvent extracts from the whole fruit or exocarp of *S. pinnata* have previously been reported to have antimicrobial activity against a wide variety of microbial strains [14,35,36]. The fruit essential oil of *S. pinnata* from Egypt had antibacterial potential, but showed no activity against *A. fumigatus* and *C. albicans* [25], while our study demonstrated strong activity against these two fungi by the essential oil from the fruit peel. This antimicrobial activity might be due to the high content of furfural, α-terpineol, and γ-terpineol, which could inhibit the proliferation of *Salmonella bacteria* and *Bacillus subtilis* [37], have showed a positive effect on the reduction of microbes in a room [38], have antifungal potential against one of the main postharvest pathogens in citrus, *Geotrichum citri-aurantii* [39], and have been reported to have antimicrobial activity against *Streptococcus mutans*, *Propionibacterium acnes*, *Proteus vulgaris*, *Saccharomyces cerevisiae*, *Candida utilis*, and *Pityrosporum ovale* with MICs arranging from 50 to 800 µg/mL, respectively [40]. Furthermore, this result matched the traditional use of *S. pinnata* as an indigenous remedy for diarrhea, digestive disorders, and whooping cough, which might be caused by *Staphylococcus aureus* [41] and *A. fumigatus* [42].

### 2.4. Anti-Inflammatory Activity

#### 2.4.1. Effect of Essential Oil on Cell Viability

**Figure 3:**
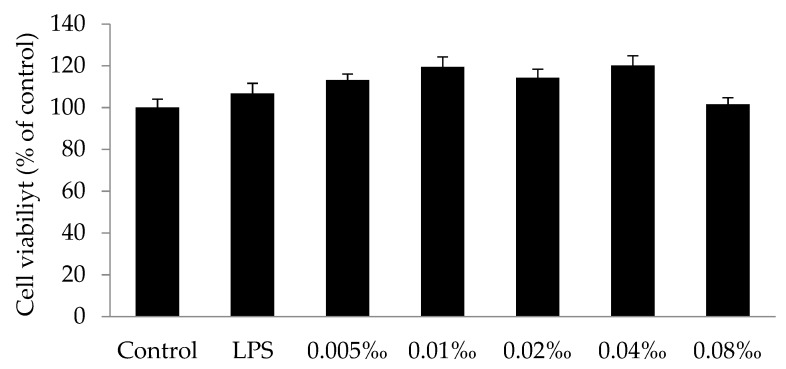
Effect of the essential oil of *Spondias pinnata* (EOSP) on the viability of RAW 264.7 cells by MTS assay. Results were expressed as a percentage of MTS production by control cells maintained in culture medium. Values are presented as mean ± standard deviation (SD) of three independent tests.

The cells were maintained in culture medium (control) or were incubated with 1 μg/mL lipopolysaccharide (LPS), or with the essential oils of *Spondias pinnata* (EOSP) (0.005–0.08‰) in the presence of 1 μg/mL LPS for 24 h. The essential oil showed no significant cytotoxic activity compared to the control on LPS-introduced RAW 264.7 cells at concentrations of up to 0.08‰, as assessed by the Dunnett’s multiple comparison test (Figure 3). We therefore inferred that concentrations of up to 0.08‰ of the total essential oil could be safe for further development of this essential oil in the food, pharmaceutical, and cosmetic industries.

#### 2.4.2. Effect of Essential Oil on NO Production

**Figure 4:**
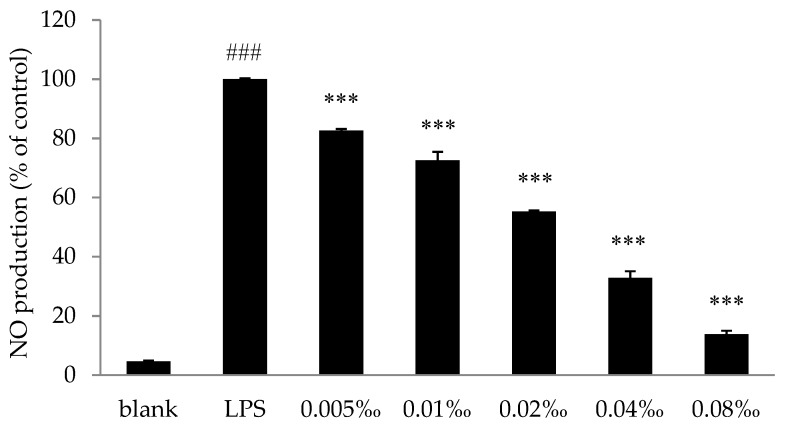
Effect of the essential oil of *Spondias pinnata* (EOSP) on NO production in RAW 264.7 cells. Results were expressed as percentages of NO by control cells maintained in culture medium. Values are presented as mean ± standard deviation (SD) of three independent tests. ^###^ *p* < 0.001 for LPS versus the blank group. *** *p* < 0.001 for cells treated with essential oil samples versus the control group (LPS).

The effect of essential oil of *Spondias pinnata* (EOSP) samples on nitric oxide (NO) production in LPS-introduced RAW 264.7 cells was determined by the Griess reagent system. The cells were either maintained in culture medium, incubated with 1 μg/mL LPS (control), or were incubated with essential oils (0.005‰–0.08‰) in the presence of 1 μg/mL LPS for 24 h. LPS treatment significantly increased NO production compared with untreated cells (Figure 4). As assessed by the LSD test, EOSP significantly reduced NO production by 17.36%, 27.47%, 44.71%, 67.16%, and 86.19% compared with LPS treated cells at concentrations of 0.005‰, 0.01‰, 0.02‰, 0.04‰, and 0.08‰, respectively. The IC_50_ value, calculated as the concentration of EOSP that could inhibit 50% of NO production in RAW 264.7 cells, was found to be 0.031‰ ± 0.0045‰ by Probit regression analysis. The anti-inflammatory activity of EOSP can probably be attributed to the high content of α-terpineol and methyl salicylate, which have been demonstrated to have strong anti-inflammatory effects in the model of carrageenan-induced pleurisy in mouse and the LPS-induced nitrite production in murine macrophages [43], on isolated tracheal smooth muscle in guinea pig [44], and in epithelial buccal cells by inhibiting the gene expression of the IL-6 receptor [45] and through inhibiting the production of pro-inflammatory cytokines, NO, and reactive oxygen species (ROS) [46]. NO is one of the key determinants in infection, inflammation, and cancer [47], so the significant NO inhibition effect supports the use of *S. pinnata* as an indigenous remedy for inflammation, detoxification, and relief of swelling and pain.

## 3. Materials and Methods

### 3.1. Chemicals and Reagents

Standard Mueller–Hinton agar and broth (MHA and MHB) and Sabouraud agar and broth (SA and SB) were obtained from Tianhe Microbial Agents Company (Hangzhou, China). Dimethylsulphoxide (DMSO), fetal bovine serum (FBS), penicillin-streptomycin, L-glutamine, and lipopolysaccharide (LPS) were obtained from Sigma-Aldrich (St. Louis, MO, USA). Dulbecco’s modified Eagle’s medium (DMEM) was obtained from Thermo Scientific (Logan, UT, USA). The CellTiter 96 AQueous One Solution Reagent for MTS assay and Griess reagent system for NO measurement were obtained from Promega Corporation (Madison, WI, USA). All reagents were analytically standard.

### 3.2. Plant Materials and Essential Oil Extraction

The ripe fruits of *Spondias pinnata* (Linn. f.) Kurz were collected from several trees in the Xishuangbanna Tropical Botanical Garden (XTBG), Chinese Academy of Science (located in Menglun Township, Yunnan province, China). A voucher specimen (no. 029891) was deposited in the XTBG herbarium (HITBC). The air-dried fruit peels (300 g) were minced by laboratory mill and subjected to steam distillation extraction using 2000 mL de-ionized water for 4 h. The essential oil was extracted by Likens and Nickerson designed simultaneous distillation extraction (SDE) method [48]. In brief, diethyl ether (30 mL) was used as the solvent to extract the essential oil while the sample was simultaneously steam distilled. The essential oil and solvent were collected together and then the diethyl ether was removed using a rotary evaporator to yield the essential oil at room temperature and room pressure. The collected essential oil was stored at −18 °C in the dark for further use.

### 3.3. Gas Chromatography/Mass Spectrometry (GC-MS) Analysis

The analysis of the essential oil sample was performed using an Agilent Technologies 7890A GC, equipped with an HP-5 MS capillary column (30 m × 0.25 mm; film thickness, 0.25 µm) and a mass spectrometer 5975C of the same company as the detector. MS were taken at 70 eV with a mass range of *m*/*z* 45–500. Helium was used as the carrier gas at a flow rate of 1 mL/min. Injector and detector (MS transfer line) temperatures were both 250 °C. Column temperature was gradually increased from 40 °C to 160 °C at 3 °C/min and increased to 250 °C at 20 °C/min, then held for 10 min. Lastly, 0.2 µL of the diluted sample was injected manually.

### 3.4. Identification of the Components

The experiment GC retention indices (RI) were determined with reference to homologous series of n-alkanes C_7_–C_30_ under identical experimental conditions and the reported RI equation [49]. Then, the components were identified by comparing calculated experimental GC retention indices with the GC retention indices reported in the National Institute of Standards and Technology (NIST)Chemistry WebBook [50], by matching their mass spectra with those recorded in the NIST 08 database (National Institute of Standards and Technology, Gaithersburg, MD, USA) and mass spectra with published data. The percentage composition of individual components was computed by the normalization method from the GC peak areas, assuming an identical mass response factor for all compounds.

### 3.5. Cytotoxicity Assays

Five tumor cell lines (HL-60 human myeloid leukemia, SMMC-7721 hepatocellular carcinoma, A-549 lung cancer, MCF-7 breast cancer, and SW480 colon cancer) were used for the cytotoxicity assay and were cultured in (Roswell Park Memorial Institute)RPMI-1640 (Hyclone, South Logan, UT, USA), supplemented with 10% fetal bovine serum (Hyclone), in 5% CO_2_ at 37 °C. The cytotoxicity assay was conducted by the MTS (3-(4, 5-dimethylthiazol-2-yl)-5(3-carboxymethoxyphenyl)-2-(4-sulfopheny)-2H-tetrazolim) method in 96-well microplates with cisplatin (Sigma) as the positive control. The IC_50_ values were calculated by the Reed and Muench method [51].

### 3.6. Antimicrobial Activity Assay

#### 3.6.1. Microbial Strains and Culture Media

The essential oils were individually tested against 7 microbial strains. *Staphylococcus aureus* (ATCC 25923), *Escherichia coli* (ATCC 25922), *Pseudomonas aeruginosa* (ATCC 27853), and *Candida albicans* (ATCC Y0109) were provided from the National Institute for the Control of Pharmaceutical and Biological Products (NICPBP, China). *Aspergillus fumigatus*, *Acinetobacter baumannii*, and *Klebsiella pneumonia* were isolated and provided by Kunming General Hospital, PLA, Kunming, China. Standard Mueller–Hinton agar and broth (MHA and MHB) and Sabouraud agar and broth (SA and SB) were used as the bacterial and fungal culture media, respectively.

#### 3.6.2. Minimal Inhibitory, Bactericidal, and Fungicidal Concentration (MIC, MBC, and MFC) Assay

MIC and MBC/MFC were evaluated against tested sensitive microbial strains by standardized broth microdilution methods according to Clinical and Laboratory Standards Institute (CLSI) guidelines [52,53,54,55] and the procedures reported previously [56]. The MICs, MBCs, and MFCs were determined with starting inoculums of 1.0 × 10^6^ (colony-forming unit) CFU/mL for bacteria and 1.0 × 10^4^ CFU/mL for fungi, incubated at 35 °C for 24 h, and were examined for growth in daylight. For the MIC assay, the essential oil was dissolved in dimethylsulfoxide (DMSO) and sterilized by filtration through 0.45 µM Millipore filters. Then, 100 µL of an appropriate medium, essential oil solutions, and inoculums were dispensed onto a 96-well plate. MIC values were the lowest concentration of a given extract that completely inhibited the visible microbial growth. For the MBC/MFC assay, 10 µL samples taken from the clear wells of the microbroth susceptibility studies were placed onto the surfaces of MHA or SA plates to determine MBC/MFC. The MBC/MFC was the concentration at which a 99.9% reduction of the original inoculum was observed [52,53]. The MICs, MBCs, and MFCs of amikacin, fluconazole, vancomycin, tigecycline, ciprofloxacin, and cefotaxime were also determined in parallel experiments in order to control for the sensitivity of the standard test organisms. The culture medium and solvent (DMSO) were employed as negative controls and their values were deducted accordingly. All tests were performed in triplicate.

### 3.7. Anti-Inflammatory Activity Assay

#### 3.7.1. Cell Culture

The murine macrophage cell line RAW 264.7 was obtained from Kunming Institute of Zoology, Chinese Academy of Sciences (KCB200603YJ) and maintained in DMEM containing 10% fetal bovine serum, 1% penicillin-streptomycin, and 1% L-glutamine at 37 °C in a 5% CO_2_ incubator (Thermo Scientific, Forma371, Steri-cycle, Waltham, MA, USA) and was sub-cultured every 2 days.

#### 3.7.2. Cell Viability Assay

Cell viability was evaluated by the MTS assay as reported [57]. In the MTS assay, 100 μL cell suspensions (1 × 10^5^ cells/well) were cultivated in 96-cell plates for 18 h, as described. Then cells were pre-treated with various concentrations of the essential oils for 30 min before they were further incubated in the presence of 1 μg/mL LPS for 24 h. Finally, 20 µL of CellTiter 96 AQueous One Solution Reagent, prepared by MTS (3-[4,5,dimethylthiazol-2-yl]-5-[3-carboxymethoxy- phenyl]-2- [4-sulfophenyl]-2H-tetrazolium, inner salt) in the presence of phenazine ethosulfate (PES), was added to each well and incubated for 1 h at 37 °C in the 5% CO_2_ incubator. The absorbance of each well was measured at 490 nm directly using a multifunctional microplate reader (Thermo Scientific VarioSkan Flash, USA). Results were expressed as a percentage of MTS production by control cells maintained in culture medium. Values were performed in triplicate and presented as mean ± standard deviation (SD).

#### 3.7.3. Measurement of NO Production

Nitric oxide (NO) production by LPS-stimulated Raw 264.7 cells was measured by the accumulation of nitrite in the culture supernatants, using the Griess reagent system (Promega, Madison, WI, USA) following the manufacturer’s instructions. Cells were seeded in 96-well plates at 1 × 10^6^ cells/well and then incubated with culture medium (control) for 18 h. Then cells were pretreated with various concentrations of the essential oils for 30 min before they were stimulated with 1 μg/mL LPS and further cultured for an additional 24 h. In brief, 50 μL of culture supernatants were collected and mixed with the Griess reagent system (1% sulfanilamide in 0.1 mol/L HCl and 0.1% N-(1-naphthyl) ethylenediamine dihydrochloride). Then, the plates were incubated at room temperature in shade for 10 min. The absorbance was measured at 550 nm in a multifunctional microplate reader (Thermo Scientific VarioSkan Flash, USA). Nitrite concentration was determined from a sodium nitrite standard curve. Results were expressed as a percentage of NO produced by control cells maintained in culture medium. Values were presented as mean ± standard deviation (SD) of three independent tests.

### 3.8. Statistical Analysis.

All experiments were performed in triplicate and expressed as mean values ± standard deviation (SD). The 50% inhibition concentration (IC_50_) was calculated by Probit regression analysis using SPSS 17.0 for Windows (SPSS Inc. Chicago, IL, USA). One-way ANOVA analysis, with a Dunnett’s multiple comparison test and least significant difference (LSD) test, was performed using SPSS 17.0 for statistical evaluation. Differences were accepted as significant at *p* < 0.05.

## 4. Conclusions

The results demonstrated that the fruit peel essential oil of *S. pinnata* (EOSP) has antimicrobial activity against five pathogenic strains and anti-inflammatory property by significant inhibition of NO production in LPS-stimulated RAW 264.7 cells. The results support the continued use of *S. pinnata* as a nutritional fruit and medicinal ingredient in China and in other South and East Asian countries. In addition, the fruit peels might be further applied as a new natural antimicrobial and anti-inflammatory ingredient in the medicine and food industries.

## Figures and Tables

**Table 1:** Chemical composition of the essential oil from the fruit peel of *Spondias pinnata*.

No	RT	RI ^cal^	RI ^lit^	Compound	Area (%)
1	5.34	799	800	Hexanal	0.81
2	6.32	830	835	Furfural	17.14
3	6.96	850	853	2-Hexenal	4.17
4	7.09	854	855	(*Z*)-3-Hexen-1-ol,	4.88
5	9.16	914	918	Ketone, 2-furyl methyl	0.22
6	9.98	932	930	α-Pinene	0.93
7	10.58	946	945	Camphene	0.21
8	11.38	964	964	5-Methyl-2-furaldehyde	0.57
9	11.81	974	978	β-Pinene	0.19
10	12.58	991	993	β-Myrcene	0.33
11	13.10	1003	1005	3-Carene	0.40
12	13.34	1008	1008	(3*E*)-Hexenyl acetate	0.28
13	13.60	1014	1016	Isocineole	0.89
14	14.05	1023	1025	p-Cymene	0.32
15	14.22	1027	1028	Limonene	2.04
16	14.99	1043	1043	Benzeneacetaldehyde	0.76
17	15.23	1048	1048	(*E*)-β-Ocimene	0.99
18	15.67	1058	1060	γ-Terpinene	0.58
19	16.31	1072	1068	Linalool oxide	4.38
20	16.67	1079	1078	2-Furaldehyde diethyl acetal	0.39
21	17.06	1087	1088	(*E*)-Linalool oxide, furanoid	5.33
22	17.41	1095	1092	Benzoic acid, methyl ester	1.25
23	17.68	1101	1103	Linalool	1.45
24	17.88	1105	1105	Nonanal	1.28
25	18.24	1112	1110	2-Fenchanol	0.68
26	19.25	1134	1138	1-Terpineol	0.52
27	19.74	1144	1149	β-Terpineol	0.94
28	20.19	1154	1153	Ocimenol	0.44
29	20.71	1164	1163	Isoborneol	1.04
30	20.99	1170	1072	Ethyl benzoate	9.05
31	21.25	1176	1177	Terpinen-4-ol	2.66
32	21.92	1190	1190	α-Terpineol	13.09
33	22.08	1193	1197	Methyl salicylate	5.88
34	22.22	1196	1201	γ-Terpineol	5.55
35	25.23	1262	1260	(*E*)-2-Decenal	0.78
36	30.57	1382	1388	β-(*E*)-Damascenone	0.26
37	32.00	1416	1418	Caryophyllene	0.33
38	34.04	1467	1462	Ethyl cinnamate	3.55
39	39.90	1637	1635	γ-Eudesmole	0.17
40	45.46	1996	1999	Ethyl hexadecanoate	0.44
Total identified					95.19
Aliphatic alcohols					39.42
Monoterpene hydrocarbons					29.62
Aromatics					22.03
Oxygenated monoterpenes					3.62
Sesquiterpene hydrocarbons					0.50

RT refers to retention time. RI ^cal^ refers to the retention index experimentally calculated using C7-C30 alkanes. RI ^lit^ refers to the retention index taken from the National Institute of Standards and Technology (NIST) database.

**Table 2:** Cytotoxic activity (IC_50_ µg/mL) of the essential oil of *Spondias pinnata* (EOSP) for cancer cell lines.

Compound	HL-60	SMMC-7721	A-549	MCF-7	SW480
EOSP	13.29	44.67	34.43	48.60	50.21
Cisplatin	0.50	2.08	2.22	3.25	2.97

HL-60, SMMC-7721, A-549, MCF-7 and SW480 represent human myeloid leukemia, hepatocellular carcinoma, lung cancer, breast cancer, colon cancer respectively.

**Table 3:** MIC and MBC/MFC (µg/mL) of the essential oil of *Spondias pinnata* (EOSP) ^a^.

Microbial Strain		EOSP		Positive Control ^b^	
**Gram-positive bacteria**		**MIC**	**MBC/MFC**	**MIC**	**MBC/MFC**
	*S. aureus*	512	512	0.25	0.5
**Gram-negative bacteria**	*A. baumannii*	512	>512	0.5	1
	*E. coli*	>512	ND	0.05	0.25
	*K. pneumonia*	>512	ND	256	512
	*P. aeruginosa*	128	128	0.25	1
**Fungi**	*A. fumigatus*	16	32	512	1024
	*C. albicans*	128	256	0.5	1

^a^ All tests were performed in triplicate. ^b^ Positive control: Vancomycin for *S. aureus*; Fluconazole for *A. baumannii* and *C. albicans*; Cefotaxime for *E. coli*; Amikacin for *K. pneumonia*; Ciprofloxacin for *P. aeruginosa*; Tigecycline for *A. fumigatus*. ND means not determined.
